# The Weather Cock

**Published:** 1903-12

**Authors:** 


### THE WEATHER COCK.

We are glad of the opportunity presented by Dr. Rheem's letter for two reasons: First, that we may withdraw our second-hand statement which we do, and, second, that we may set Dr. Rheem and all others right as to our position on the question of osteopathy and all kindred things. We believe in all of them. Our motto: "The smallest possible quantity of the best obtainable means to produce a desired therapeutic result." Whether it be an alkaloidal granule, a mustard-paste, an osteopathic manipulation, a therapeutic suggestion or a Christian Science séance, we don't care a rap as long as it comes under the above, by which we stand, and is really the best thing. Personally, we have enjoyed and profited by osteopathic treatment. We know we have profited by suggestive therapeutics. We try to be both Christian and scientific. We are hydropathists to the extent of believing in cold water internally, externally and eternally, and we are "cureopaths" to the extent of believing it right to cure one's patients or, perhaps better, to help them to get well in the simplest, shortest way possible. Any votary of the healing art that is satisfied with our platform can stand with us shoulder to shoulder. If he isn't satisfied, that's quite another thing; but we have yet to see the "healer" who on serious thought wasn't squarely with us. What's the use of "scrapping"?

These editorial comments on a subscriber's letter in the *Alkaloidal Clinic* breathe a spirit of liberality that is fine and bracing and serve probably to square the *Alkaloidal Clinic* with all kinds of odds and ends of doctors and sell many granules. But must an editor always be all things to all people and turn with every breeze like the battered chanticleer on top of the barn, just because he is an editor? Is not he entitled to a few opinions and convictions of his own? Must he divide his gods up to suit all worshipers, into a polytheism truly Egyptian? Think of the *lucus a non lucendo* of a man agreed with every other man and no two men believing the same thing. The world would be in a state of chaos beside which a national convention of the Daughters of the American Revolution would be as quiet as a cabinet meeting. You are too much of an expansionist, Brother Abbott; take a few W. A. antiseptic granules and quiet the borborygmus and reduce your girth.

ATLANTA MEDICAL LIBRARY ASSOCIATION held its annual meeting in November. The society was in flourishing condition as shown by the treasurer's report. Many books had been donated during the year and the leading medical journals in English subscribed for. If every medical man in Atlanta would display an interest in this society there could soon be built up a library that would be a pride to the profession as well as an aid of inestimable value. The following officers were elected for the ensuing year: Dr. Michael Hoke, President; Dr. V. O. Hardon, Vice-President; Dr. E. Bates Block, Secretary and Treasurer; Dr. A. W. Sterling, Dr. W. S. Elkin, Dr. Bernard Wolff, Executive Committee.

---

THE Atlanta Society of Medicine was delightfully entertained by Dr. V. O. Hardon, at his residence, 283 Peachtree street, on Wednesday evening, November 3. Musical selections were rendered by Mrs. Hardon, Mrs. Dr. Sterling, Dr. Frank Boland and Dr. E. C. Cartledge. A dutch supper was served together with the German national beverage—both of which met with instant and hearty appreciation. Social gatherings of this description add immensely to professional *esprit de corps*, and the example set by Dr. Sterling and Dr. Hardon should be followed.

---

MR. W. E. PELHAM, JR., PH.G., the junior member of the noted wholesale and retail drug and sundry firm, of Newberry, S. C., is now attending his third term at the Medical Department of Tulane University, New Orleans. He is already a graduate of the well-known and reputable College of Pharmacy, Baltimore, and will soon be honored as W. E. Pelham, M.D., Ph.G.

---

Mr. C. P. PELHAM, of Newberry, S. C., has been on the road in the interest of H. K. Wampole & Co. the past six months, and his energies and strong efforts has gained that which he deserves, "The top of the ladder," or heads the list of salesmen. Good for a new beginner against old experienced salesmen.

